# Differences in Clinical Outcomes and Survival Among Primary, Secondary, and Concomitant Carcinoma In Situ of the Bladder

**DOI:** 10.7759/cureus.69625

**Published:** 2024-09-18

**Authors:** Shinro Hata, Hiroyuki Fujinami, Mayuka Shinohara, Shinya Sejiyama, Toru Inoue, Hiromitsu Mimata, Toshitaka Shin

**Affiliations:** 1 Department of Urology, Faculty of Medicine, Oita University, Yufu, JPN

**Keywords:** bacillus calmette–guérin, bladder cancer, carcinoma in situ, transurethral resection of bladder tumor (turbt), urothelial carcinoma

## Abstract

Purpose: Carcinoma in situ (CIS) is a flat, high-grade, and aggressive form of urothelial carcinoma with a high risk of progression to muscle-invasive disease and metastasis. This study aimed to investigate differences in clinical outcomes and survival among patients with primary, secondary, and concomitant CIS of the bladder.

Methods: A total of 209 patients diagnosed with CIS between 2010 and 2022 in our department with a minimum follow-up of 12 months were retrospectively analyzed. Patients with muscle-invasive cancer at diagnosis, those with recurrence within one month after diagnosis, and those with primary malignant melanoma were excluded. The recurrence, progression, and cancer-specific mortality rates of patients receiving Bacillus Calmette-Guérin therapy for CIS were analyzed.

Results: A total of 96 patients with primary (*n* = 18), secondary (*n* = 29), and concomitant CIS (*n* = 49) were included in the analysis. The median follow-up was 52.2 months. Patients with secondary CIS had a significantly higher recurrence rate than those with concomitant CIS (58.6% vs. 32.7%, *p* = 0.016). However, no significant difference in progression rates was observed among the three groups. Furthermore, no significant association was observed between CIS subtypes and recurrence-free survival (RFS) (HR = 1.45, 95% CI 0.96-2.46, *p* = 0.16) or progression-free survival (PFS) (HR = 2.20, 95% CI 0.99-4.87, *p* = 0.054).

Conclusion: Secondary CIS had a significantly higher recurrence rate than concomitant CIS. However, no statistically significant association was observed between CIS subtypes and RFS or PFS.

## Introduction

Bladder cancer is a significant global health concern and the 10th most commonly diagnosed cancer worldwide, with approximately 573,000 new cases and 213,000 deaths reported in 2020 [1]. Extensive research efforts have been made to understand the pathogenesis and management of bladder cancer. However, critical knowledge gaps persist regarding the role of carcinoma in situ (CIS) in the spectrum of bladder cancer. CIS is a flat, high-grade urothelial lesion confined to the mucosa and is considered a precursor lesion with a high risk of progression to invasive bladder cancer [2]. CIS has been reported to be associated with a significant increase in the risk of disease recurrence and progression to muscle-invasive bladder cancer, highlighting the need for a better understanding of CIS [3]. Despite the recognized importance of CIS in the context of bladder cancer, differences in clinical outcomes and survival among the various subtypes of CIS are not fully elucidated. Primary CIS, which presents as a de novo lesion without prior or concurrent papillary tumors, has been reported to have a more aggressive clinical course than secondary CIS, which develops after a history of papillary tumors [4]. However, the prognostic significance of concomitant CIS, which is diagnosed simultaneously with papillary tumors, remains poorly understood. Moreover, the effects of these CIS subtypes on recurrence and progression rates following intravesical Bacillus Calmette-Guérin therapy, the standard treatment for high-risk nonmuscle-invasive bladder cancer, have not been thoroughly investigated [5]. These knowledge gaps highlight the need for a comprehensive evaluation of the clinical behavior and outcomes associated with primary, secondary, and concomitant CIS to inform risk stratification and develop personalized management strategies.

Therefore, this study aimed to investigate differences in clinical outcomes and survival among patients with primary, secondary, and concomitant CIS of the bladder and to address the existing knowledge gaps by retrospectively analyzing a cohort of patients diagnosed with CIS at our institution over 12 years. We aimed to provide insights into the prognostic implications of CIS presentation timing by comparing recurrence, progression, and cancer-specific mortality rates among CIS subtypes. The findings of this study contribute to a better understanding of the clinical behavior of bladder CIS subtypes and provide a foundation for future investigations that may help reveal the role of CIS in the spectrum of bladder cancer.

## Materials and methods

Study design and patient selection

This was a retrospective study including patients diagnosed with CIS of the bladder between 2010 and 2022 in our department. The inclusion criteria were patients with primary, secondary, and concomitant CIS with a minimum follow-up period of 12 months. The exclusion criteria were patients with muscle-invasive cancer at diagnosis, those with recurrence within one month after diagnosis, and those with primary malignant melanoma. This study was reviewed and approved by the Institutional Review Board of Oita University (approval number: 2358). Informed consent was obtained from all patients through websites using the opt-out method.

Data collection

Data were collected from electronic medical records, including demographics, clinical history, pathology reports, and treatment details. The collected data included age at diagnosis, gender, presenting symptoms, tumor characteristics, treatment modalities, recurrence, progression, and survival outcomes.

Definitions of CIS subtypes

Primary CIS was defined as an isolated CIS lesion with no previous or concurrent papillary tumor. Secondary CIS was defined as a CIS lesion detected during the follow-up of a previous papillary tumor. Concomitant CIS was defined as CIS detected simultaneously with a papillary tumor.

Follow-up

Patients were followed up with cystoscopy and urine cytology every three to six months for the first two years, every six to 12 months for the next three years, and annually thereafter according to standard institutional protocols. CT scans were performed every six to 12 months. Follow-up data were collected until the last visit or death.

Outcome measures

Recurrence-free survival (RFS) and progression-free survival (PFS) were analyzed using Kaplan-Meier curves and log-rank tests. Recurrence was defined as the reappearance of CIS or the development of a new papillary tumor after the initial treatment. Progression was defined as the development of a muscle-invasive disease or metastasis. Additionally, cancer-specific mortality rates and factors associated with recurrence or progression were analyzed.

Statistical analysis

Patient characteristics and clinical outcomes were summarized using descriptive statistics. Continuous variables were expressed as medians and ranges, whereas categorical variables were presented as frequencies and percentages. RFS and PFS were estimated using Kaplan-Meier curves, and differences between CIS subtypes were assessed using log-rank tests. Multivariate Cox proportional hazards regression analysis was performed to identify factors associated with RFS and PFS. A p-value <0.05 indicated statistical significance. All statistical analyses were performed using EZR (Saitama Medical Center, Jichi Medical University, Saitama, Japan), a graphical user interface for R (The R Foundation for Statistical Computing, Vienna, Austria). More precisely, it is a modified version of R Commander designed to add statistical functions frequently used in biostatistics [6].

## Results

Patient characteristics

**Figure 1:**
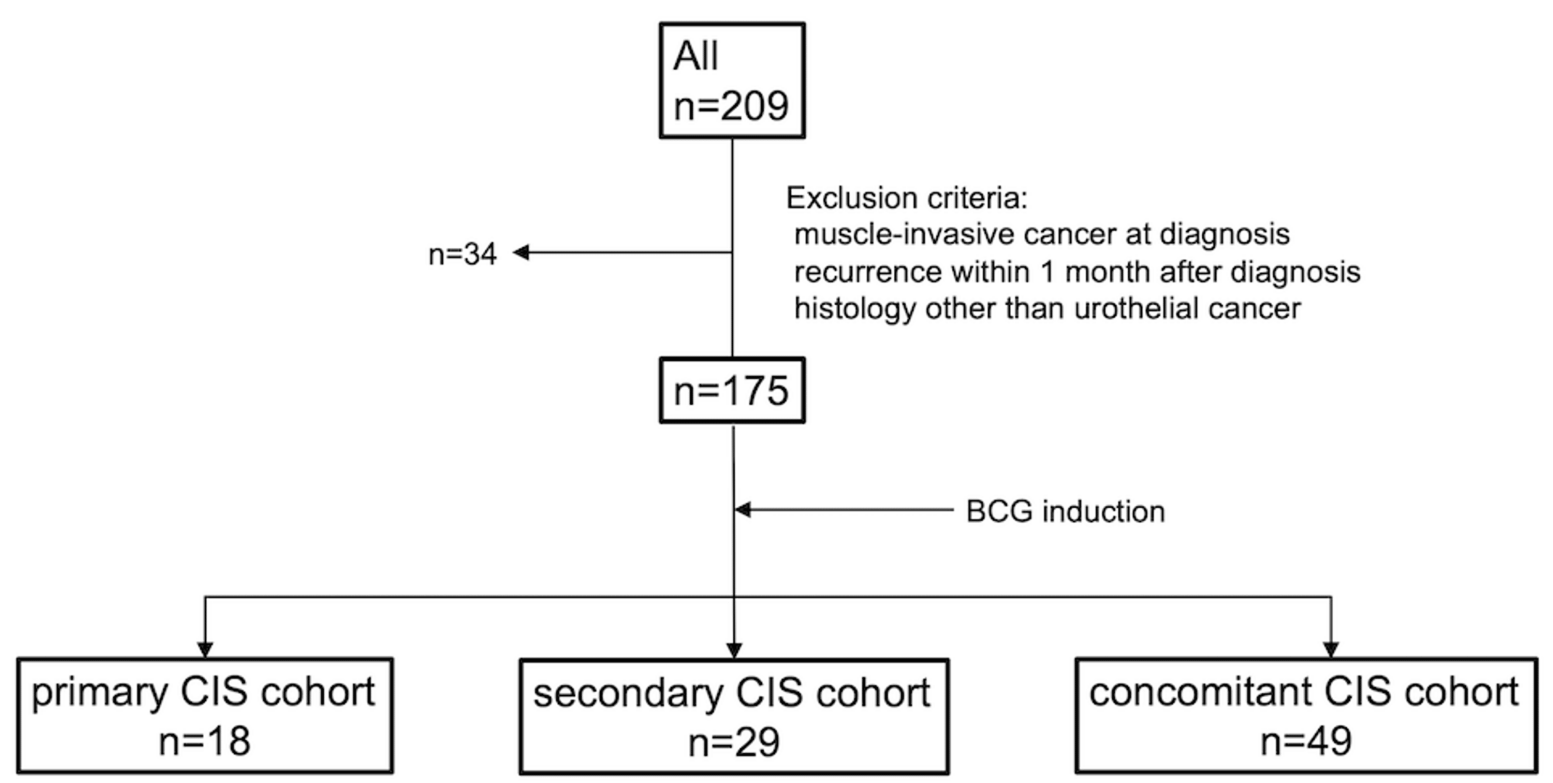
Flowchart of the study CIS: carcinoma in situ, BGC: Bacillus Calmette-Guerin

A total of 209 patients with CIS of the bladder were enrolled in this study. Of the 209 patients, 34 were excluded due to muscle-invasive cancer at diagnosis (n = 25), recurrence within one month after diagnosis (n = 8), and primary malignant melanoma (n = 1). Finally, 96 patients with primary (n = 18, 18.8%), secondary (n = 29, 30.2%), and concomitant CIS (n = 49, 51.0%) treated with Bacillus Calmette-Guérin were included in the analysis (Figure 1).

The median age at diagnosis was 74 years. Of the 96 patients, 81 (84.4%) were men, and 15 (15.6%) were women. The median follow-up period was 52.2 months (Table 1).

**Table 1:** Characteristics of the study population IQR: interquartile range, RFS: recurrence-free survival, PFS: progression-free survival

Variables	All (n = 96)	Primary (n = 18)	Secondary (n = 29)	Concomitant (n = 49)
Gender, n (%) (M/F)	81 (84.4)/15 (15.6)	13 (72.2)/5 (27.8)	26 (89.7)/3 (10.3)	42 (85.7)/7 (14.3)
Age, median (IQR)	74 (70-80)	77 (74-81)	74 (67-76)	73 (70-80)
Observation time, median (IQR), month	52.2 (26.9-82.6)	43.5 (31.1-80.0)	66.5 (40.6-120.2)	49.7 (22.6-73.0)
Recurrence, n (%)	39 (40.6)	6 (33.3)	17 (58.6)	16 (32.7)
Progression, n (%)	17 (17.7)	2 (11.1)	7 (24.1)	8 (16.3)
Cancer-specific mortality, n (%)	8 (8.3)	2 (11.1)	5 (17.2)	1 (2.0)
RFS, median (IQR), month	27.5 (12.7-68.0)	34.6 (16.4-64.9)	24.2 (9.1-97.0)	28.4 (11.8-61.2)
PFS, median (IQR), month	46.1 (17.9-73.5)	43.0 (26.8-68.4)	48.3 (16.4-106.1)	46.8 (16.2-69.6)

Recurrence and progression rates

During the follow-up period, recurrence was observed in six (33.3%), 17 (58.6%), and 16 (32.7%) patients with primary, secondary, and concomitant CIS, respectively. Patients with secondary CIS had a significantly higher recurrence rate than those with concomitant CIS (p = 0.016). Progression was observed in two (11.1%), seven (24.1%), and eight (16.3%) patients with primary, secondary, and concomitant CIS, respectively. No significant differences in progression rates were observed among patients with primary, secondary, and concomitant CIS (p > 0.05) (Table 2).

**Table 2:** P-values of recurrence and progression rates among CIS subtypes CIS: carcinoma in situ

	Primary vs. secondary	Primary vs. concomitant	Secondary vs. concomitant
Recurrence	0.073	0.96	0.016
Progression	0.26	0.60	0.36

Survival analysis

**Figure 2:**
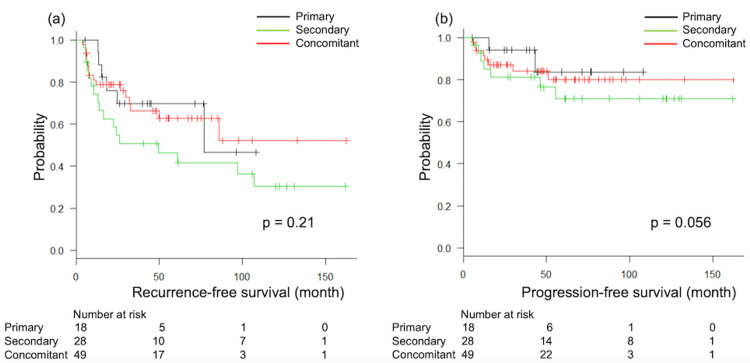
Kaplan-Meier curves for RFS (a) and PFS (b) according to CIS subtype RFS: recurrence-free survival, PFS: progression-free survival, CIS: carcinoma in situ

Kaplan-Meier analysis revealed no significant differences in RFS (p = 0.21) or PFS (p = 0.56) among patients with primary, secondary, and concomitant CIS subtypes. The median RFS was 77.0 months (95% confidence interval (CI) 18.2-NA) for patients with primary CIS, 49.4 months (95% CI 13.0-107.0) for patients with secondary CIS, and not reached (95% CI 33.0-NA) for patients with concomitant CIS. The median PFS was not reached for patients with any CIS subtype (Figure 2).

Multivariate analysis

The multivariate Cox proportional hazards analysis revealed no statistically significant associations between CIS subtypes and RFS (hazard ratio (HR) = 1.45, 95% CI 0.96-2.46, p = 0.16) or PFS (HR = 2.20, 95% CI 0.99-4.87, p = 0.054) (Tables 3-4). However, male gender was associated with worse PFS (Table 4).

**Table 3:** Multivariable Cox regression analyses for the prediction of RFS CIS: carcinoma in situ

Variables	Hazard ratio	95% confidence interval	p-value
CIS subtypes	1.45	0.96-2.46	0.16
Age ≥75 years	2.26	0.85-6.06	0.10

**Table 4:** Multivariable Cox regression analyses for the prediction of PFS CIS: carcinoma in situ

Variables	Hazard ratio	95% confidence interval	p-value
CIS subtypes	2.20	0.99-4.87	0.054
Age ≥75 years	1.67	0.46-6.07	0.44
Gender	4.24	1.38-13.09	0.012
Positive urinary cytology	1.70	0.74-3.93	0.21

## Discussion

This study investigated differences in clinical outcomes and survival among patients with primary, secondary, and concomitant CIS of the bladder. The findings reveal two key insights. First, secondary CIS demonstrated a significantly higher recurrence rate than concomitant CIS, whereas no significant differences in progression were observed among the three groups. Second, Cox proportional hazards analysis revealed that the CIS subtype was not significantly associated with RFS or PFS. These findings contribute to our understanding of the clinical behaviors of different CIS subtypes and their potential implications for the risk stratification and management of bladder cancer.

The higher recurrence rate in secondary CIS compared with that in concomitant CIS is a notable finding that warrants further discussion. This study revealed that 58.6% of patients with secondary CIS and 32.7% of patients with concomitant CIS experienced recurrence, showing a statistically significant difference (p = 0.016). These findings are consistent with those of a previous study by Sylvester et al. [3], which showed that patients with a history of tumors were at higher risk of recurrence. The increased recurrence rate in secondary CIS may be attributed to the field cancerization effect, where the entire urothelium is at risk for malignant transformation due to prolonged exposure to carcinogens [2]. Interestingly, although recurrence rates differed, no significant differences in progression rates were observed among the three CIS subtypes. This finding contradicts the notion that secondary CIS is necessarily associated with a more aggressive disease course. The implications of these findings are significant for clinical practice, suggesting that although patients with secondary CIS may require more careful surveillance for recurrence, the risk of progression may not be substantially different between patients with secondary CIS and those with primary or concomitant CIS [2].

The Cox proportional hazards analysis revealed that the CIS subtype was not significantly associated with RFS or PFS. Specifically, the analysis revealed that the CIS subtype was not an independent predictor of RFS (HR = 1.45, 95% CI 0.96-2.46, p = 0.16) or PFS (HR = 2.20, 95% CI 0.99-4.87, p = 0.054). These findings are consistent with those of previous studies [7-10]. Conversely, primary CIS has been reported to be associated with a more aggressive clinical course [4,11,12]. The findings of this study indicate that the timing of CIS presentation (primary, secondary, or concomitant) may not be as crucial in determining long-term outcomes as previously thought. This finding challenges existing paradigms in bladder cancer prognostication and suggests that other factors, such as molecular characteristics and treatment response, are more influential in determining patient outcomes [13]. The implications of this finding are significant for clinical practice, which suggests that treatment decisions and follow-up protocols should not be based solely on the CIS subtype but should consider a broader range of prognostic factors.

The findings of this study have broader implications for managing and understanding bladder CIS. Although the findings indicated that the CIS subtype may not be a strong independent predictor of long-term outcomes, they emphasized the complex nature of bladder cancer progression and the need for a multifaceted risk stratification approach [14]. These insights could influence future research directions, potentially shifting the focus toward identifying more robust prognostic markers, such as molecular subtypes and immune signatures, as suggested by recent studies [15-17]. From a clinical perspective, the findings may impact treatment decision-making processes. For instance, the higher recurrence rate observed in patients with secondary CIS warrants more intensive follow-up protocols, even if the progression risk is not significantly different. Moreover, the lack of significant differences in survival outcomes among CIS subtypes indicates that current treatment strategies, particularly Bacillus Calmette-Guérin immunotherapy, may be equally effective across these subgroups. This highlights the importance of timely and appropriate treatment for all patients with CIS, regardless of subtype classification.

This study provides valuable insights into the clinical behavior of different CIS subtypes in bladder cancer, addressing key questions about their impact on recurrence, progression, and survival outcomes. Although a higher recurrence rate was observed in patients with secondary CIS, the lack of significant differences in progression and survival outcomes among the CIS subtypes challenges the existing assumptions. These findings indicate the complex nature of bladder cancer and the need for comprehensive risk assessment beyond the CIS subtype.

This study has some limitations. This was a retrospective single-institution study, which may limit the generalizability of the results. Additionally, the study included a relatively small sample size, particularly patients with primary CIS, which may have affected our ability to detect subtle differences among subgroups. Therefore, further studies are needed to validate the findings of this study in larger, multi-center cohorts. Additionally, prospective studies incorporating molecular and genetic profiling could provide further insights into the biological mechanisms underlying the observed differences in recurrence rates and help identify more precise prognostic markers for patients with CIS.

## Conclusions

This study investigated differences in clinical outcomes and survival among patients with primary, secondary, and concomitant CIS of the bladder. The findings revealed a significantly higher recurrence rate in secondary CIS than in concomitant CIS, whereas progression rates and survival outcomes were similar across subtypes. These results challenge existing paradigms in bladder cancer management and emphasize the need for a more comprehensive risk stratification approach that incorporates molecular and genetic markers alongside traditional clinicopathological factors.
